# Clinical symptoms, endoscopic findings, and lower esophageal sphincter characteristics in patients with absent contractility

**DOI:** 10.1097/MD.0000000000031428

**Published:** 2022-10-28

**Authors:** Hang Viet Dao, Long Bao Hoang, Hue Thi Minh Luu, Hoa Lan Nguyen, Robert Joel Goldberg, Jeroan Allison, An Thi Minh Dao, Hong Thi Van Nguyen, Tomoaki Matsumura, Long Van Dao

**Affiliations:** a Internal Medicine Faculty, Hanoi Medical University, Hanoi, Vietnam; b The Institute of Gastroenterology and Hepatology, Hanoi, Vietnam; c Department of Population and Quantitative Health Sciences, University of Massachusetts Medical School, Worcester, MA, USA; d Department of Gastroenterology, Graduate School of Medicine, Chiba University, Chiba, Japan.

**Keywords:** erosive esophagitis, esophageal motility disorder, manometry

## Abstract

Absent contractility is a rare esophageal motility disorder defined by high-resolution manometry which remains poorly understood in pathogenesis and management. We investigated the clinical symptoms, upper gastrointestinal endoscopy findings, and lower esophageal sphincter (LES) characteristics in adult patients diagnosed with absent contractility on high resolution manometry and factors associated with erosive esophagitis that were found on endoscopy in these patients. A cross-sectional study was conducted in patients with absent contractility who were examined at the Institute of Gastroenterology and Hepatology, Vietnam between March 2018 and December 2020. Clinical symptoms, endoscopic findings, and LES metrics were collected and compared between individuals with and without erosive esophagitis. Logistic regression analysis was used to examine a variety of factors associated with erosive esophagitis. Among 7519 patients who underwent high resolution manometry, 204 (2.7%) were diagnosed with absent contractility. The mean age of the study sample was 45.9 years, 65.7% were women, and none had systemic sclerosis. The most common symptoms were regurgitation, belching, epigastric pain, and bloating. On endoscopy, 50% had erosive esophagitis, mostly Los Angeles grade A (42.9%). On manometry, 44.6% of the patients had LES hypotension and 68.1% had low integrated relaxation pressure in 4 seconds (IRP4s). Male sex (adjusted odds ratio = 2.01, 95% confidence interval: 1.04–3.89) and an IRP4s < 5 mm Hg (adjusted odds ratio = 2.21, 95% confidence interval: 1.12–4.37) were significantly associated with erosive esophagitis. Absent contractility was present in many patients without known systemic diseases. Erosive esophagitis was common and associated with male sex and low IRP4s.

## 1. Introduction

Absent contractility is a serious esophageal motility disorder diagnosed on high-resolution esophageal manometry which is currently considered the state-of-the-art approach to identify and confirm esophageal motility disorders. This relatively rare condition is characterized by the absence of esophageal motility and normal lower esophageal sphincter (LES) relaxation.^[1]^ Previous studies have shown that absent contractility is typically observed in patients with systemic rheumatologic diseases.^[2–4]^ Ensuring an accurate diagnosis of absent contractility is important clinically in order to optimize patient’s treatment and long-term outlook.^[2]^ However, the prevalence of absent contractility in Asian populations and its relationship with other underlying conditions and possible predisposing factors remains unknown.

Since the data of esophageal motility disorders are lacking in Asia, we conducted a cross-sectional study to investigate the clinical manifestations, endoscopic findings, and LES characteristics in patients diagnosed with absent contractility on high resolution manometry and determine factors associated with erosive esophagitis among these patients.

## 2. Methods

### 2.1. Study design

We conducted a cross-sectional study among patients who underwent high resolution manometry (HRM) between March 2018 and December 2020 at the Institute of Gastroenterology and Hepatology, Hanoi, Vietnam. Indications for performing HRM included upper gastrointestinal symptoms suggestive of esophageal motility disorders including dysphagia, regurgitation, globus sensation, non-cardiac chest pain, or extraesophageal symptoms suspected with the presence of gastroesophageal reflux disease (GERD). Eligible study patients were diagnosed with absent contractility on HRM based on the Chicago classification version 3.0 and had upper gastrointestinal endoscopic results within last 3 months.^[1]^ We excluded patients with a confirmed diagnosis of achalasia, history of gastroesophageal surgery, esophageal tumors, or current upper gastrointestinal bleeding, and those who were using drugs which affect esophageal motility (prokinetics, calcium channel blockers, nitrates, opiates, and anticholinergics).

### 2.2. Data collection

Information about patient’s medical history, clinical symptoms, endoscopy findings, and HRM test results was obtained from electronic medical records and a questionnaire completed before the HRM procedures were performed. We also asked patients whether they had been diagnosed of any systemic diseases. History of medications taken within the last 3 months were also collected, including proton pump inhibitors (PPIs), antacids, H_2_-receptor antagonists (H2RA), and prokinetics.

#### 2.2.1. Clinical symptoms.

Patient’s presenting complaints were recorded at the time they were examined. All patients were asked to complete 2 commonly used clinical questionnaire to obtain information about the presence and severity of patient’s clinical symptoms: Frequency scale for symptoms of GERD (FSSG) and GERD questionnaire (GERDQ). The FSSG is divided into two components (FSSG dysmotility score and FSSG reflux score). For both scores, the cutoff of 8 points was used to diagnose GERD.^[5,6]^

#### 2.2.2. Endoscopic findings.

The presence and severity of erosive esophagitis on endoscopy was diagnosed according to the Los Angeles classification.^[7]^ Hiatal hernia was diagnosed in the retroflex view according to the Hill classification.^[8]^ All of the procedures were performed and evaluated by endoscopists who had more than 5 years of experience in conducting and interpreting these tests.

#### 2.2.3. High resolution manometry.

All HRM procedures were performed using the Solar GI system with a 22-channel water-perfused catheter (Laborie, Poland). The catheter was nasally placed in the upright position, then the investigation was done in the supine position. Data on upper esophageal sphincter pressure, LES pressure, integrated relaxation pressure in 4 seconds (IRP4s), esophagogastric junction type, distal contractile integral (DCI), distal latency, and peristaltic break were obtained. Types of esophagogastric junction were defined based on the distance between LES and the crural diaphragm.^[9,10]^ In type I esophagogastric junction, LES and crural diaphragm completely overlap. In type II esophagogastric junction, LES and the crural diaphragm are separated by 1 to 2 cm while in type III esophagogastric junction, LES and the crural diaphragm are separated by more than 2 cm. In multi rapid swallow, contractile reverse was defined when DCI followed the last swallow ≥ 450 cm.mm Hg.s.^[11]^ We used the Chicago Classification version 3.0 to diagnose absent contractility (100% failed swallows on single wet swallows and normal IRP4s).^[1]^ The cutoff value of normal IRP4s (<19 mm Hg) was based on the instruction of manufacturer for water-perfused catheters. We also chose a cutoff for the IRP4s (<5 mm Hg vs. ≥5 mm Hg) to examine the association with erosive esophagitis; this choice was based on the median IRP4s (5.2 mm Hg) in our preliminary analysis.^[12]^

### 2.3. Statistical analysis

Patient’s characteristics were summarized as percentages and compared between patients with and without erosive esophagitis on endoscopy using the chi-square test for categorical variables and as means (standard deviation) or medians (interquartile range) for continuous variables and compared using *t* tests or Wilcoxon rank sum tests.

To examine factors associated with erosive esophagitis, univariate and multivariable logistic regression models were used. Variables included in the multivariable logistic regression model were age (≤40 years, 41–60 years, and > 60 years), sex, body mass index (underweight, normal, and overweight/obese), IRP4s < 5 mm Hg, and esophagogastric junction type. Variable selection was done through a careful review of the published literature for purposes of identifying factors that might be associated with erosive esophagitis. Data analysis was performed using SPSS version 22.0.

### 2.4. Ethical consideration

The study was approved by the Institutional Review Board of Dinh Tien Hoang Institute of Medicine under the decision No: IRB-1909 dated March 1, 2020.

## 3. Results

Between March 2018 and December 2020, a total of 7519 patients underwent HRM; of these, 204 patients (2.7%) met the manometric criteria for absent contractility. After excluding 20 patients did not have endoscopic results within the last 3 months, 184 patients were eligible.

### 3.1. Study population characteristics

The mean age of patients with HRM-confirmed absent contractility was 45.9 ± 14.6 (years), 63 patients (34.2%) were men, and 26.6% were classified as overweight. None of the study patients had known rheumatologic diseases or eosinophilic esophagitis, 5 patients (2.7%) had known history of diabetes mellitus. The most common symptoms were regurgitation (68.5%), belching (57.6%), epigastric pain (50.5%), bloating (44.6%), globus sensation (40.2%), and heartburn (40.8%). The prevalence of dysphagia and non-cardiac chest pain were 30.4% and 21.7%, respectively. The median duration of symptoms was 1 year. The percentage of patients with FSSG ≥ 8 was higher than those with GERDQ ≥ 8 (69.6% vs 38.6%). In the last 3 months, 58 patients (31.5%) were taking medications due to regurgitation symptoms (PPIs, H2-receptor antagonists, antacids, and prokinetics). Esomeprazole was the most used PPIs in our study, predominantly with the standard dose (40mg). The median duration of PPI therapy was 21 days.

On endoscopy, 50% had erosive esophagitis. The prevalence rates of Los Angeles grade A, B, and C esophagitis were 42.9%, 6.0%, and 1.1%, respectively; no patients had Los Angeles grade D esophagitis. Approximately 5% (n = 9) had a hiatal hernia on endoscopy, 5 out of 9 patients had erosive esophagitis. No endoscopy-based diagnosis of eosinophilic esophagitis was suspected.

On HRM, 44.6% of the patients (n = 82) had LES hypotension (<10 mm Hg) and 69.6% had low IRP4s (<5 mm Hg) (n = 128). The mean of IRP4s was lower in patients with esophagitis and the prevalence of low LES hypotension was also significantly higher in this group. Esophagogastric junction type I was the most common type observed. In multi rapid swallow challenge, contractile reverse (DCI ≥ 450 cm. mm Hg.s) presented in 2 patients (1.1%) and an ineffective esophageal contraction (100 ≤ DCI < 450 cm. mm Hg.s) presented in 23 patients (12.5%). Other characteristics of our patient population are presented in Table 1.

**Table 1:** Study population characteristics.

Characteristic	Esophagitis n = 92	No esophagitis n = 92	*P*-value
Male, n (%)	40 (43.5)	23 (25.0)	**.008**
Age (yrs), mean ± SD	46.7 ± 14.0	45.1 ± 15.1	.474
Age category (yrs), n (%)			.933
≤ 40	40 (43.5)	39 (42.4)	
41–60	35 (38.0)	34 (49.3)	
> 60	17 (18.5)	19 (20.7)	
Body mass index (kg/m^2^), mean ± SD	21.8 ± 2.5	21.0 ± 2.4	**.047**
BMI category, n (%)			.323
Underweight (<18.5)	10 (10.9)	11 (12.0)	
Normal (18.5 ≤ BMI < 23)	53 (57.6)	61 (66.3)	
Overweight/obese (BMI ≥ 23)	29 (31.5)	20 (21.7)	
**Clinical symptoms, n (%**)			
Regurgitation	61 (66.3)	65 (70.7)	.526
Belching	52 (56.5)	54 (58.7)	.765
Epigastric pain	44 (47.8)	49 (53.3)	.461
Bloating	40 (43.5)	42 (45.7)	.767
Globus sensation	42 (45.7)	32 (34.8)	.117
Heartburn	34 (37.0)	41 (44.6)	.294
Chronic pharyngitis	40 (43.5)	33 (35.9)	.264
Acid reflux	32 (34.8)	36 (39.1)	.541
Dysphagia	27 (29.3)	29 (31.5)	.749
Nausea	21 (22.8)	33 (35.9)	.052
Non-cardiac chest pain	20 (21.7)	20 (21.7)	1.000
Chronic cough	16 (17.4)	19 (20.7)	.598
Vomiting	8 (8.7)	14 (15.2)	.173
Weight loss	9 (9.8)	12 (13.0)	.487
Dyspnea	8 (8.7)	8 (8.7)	.982
**History of Medication**†, **n (%**)	28 (30.4)	30 (32.6)	.751
PPI ± Antacid	24 (85.7)	24 (80.0)	
PPI + H_2_-receptor antagonist	1 (3.6)	0 (0)	
PPI + Prokinetics	3 (10.7)	6 (20.0)	
**Clinical questionnaire scores**			
GERDQ score, mean ± SD	6.4 ± 3.1	7.0 ± 3.0	.191
GERDQ ≥ 8, n (%)	34 (37.0)	37 (40.2)	.650
FSSG total score, mean ± SD	12.2 ± 7.6	14.4 ± 9.1	.078
FSSG dysmotility score, mean ± SD	6.2 ± 4.7	7.2 ± 4.7	.131
FSSG reflux score, mean ± SD	6.0 ± 4.5	7.2 ± 4.7	.132
FSSG ≥ 8, n (%)	62 (67.4)	66 (71.7)	.522
**High resolution manometry**			
DCI MRS			.975
≥100 & <450 cm. mm Hg.s, n (%)	12 (13.0)	11 (12.0)	
≥ 450 cm. mm Hg.s, n (%)	1(1.1)	1 (1.1)	
Resting LESP (baseline), mean ± SD	10.7 ± 5.7	12.0 ± 5.3	.119
LESP (baseline) < 10 mm Hg, n (%)	47 (51.1%)	35 (38.0%)	.075
IRP4s, mean ± SD	3.6 ± 2.8	4.7 ± 3.0	**.018**
IRP4s < 5 mm Hg, n (%)	73 (79.3)	55 (59.8)	**.004**
Esophagogastric junction type, n (%)			
Type I	68 (74.0)	72 (78.3)	.322
Type II	12 (13.0)	6 (6.5)	
Type III	12 (13.0)	14 (15.2)	

† 58/184 patients were taken medications in the last 3 months.

BMI = body mass index, DCI MRS = distal contractile integral in multi rapid swallow, FSSG = frequency scale for the symptoms of GERD, GERDQ = gastroesophageal reflux disease questionnaire, IRP4s = 4-second integrated relaxation pressure, LESP = lower esophageal sphincter pressure, PPI = proton pump inhibitor.

*P*-values were from independent *t* tests comparing the groups with and without esophagitis for continuous variables and Chi-square tests for categorical variables; p-values in bold are statistically significant. Data are presented as n (%) or mean ± SD.

### 3.2. Factors associated with erosive esophagitis

The frequency of all examined clinical symptoms was not significantly different between individuals with and without erosive esophagitis (Table 1). Similarly, the clinical GERD scores (total FSSG score and total GERDQ score) were not significantly. After adjusting for several sociodemographic and clinical characteristics, men, and those with an IRP4s of < 5 mm Hg were associated with erosive esophagitis (Table 2).

**Table 2:** Factors associated with erosive esophagitis on endoscopy.

Characteristics	Univariate analysis	Multivariable analysis
	OR [95% CI]	OR [95% CI]
**Male**, n (%)	**2.31 [1.23–4.32]**	**2.01 [1.04–3.89]**
**Age category**, n (%)		
≤40 yrs	Reference	Reference
41–60 yrs	1.00 [0.52–1.90]	1.05 [0.52–2.14]
>60 yrs	1.15 [0.51–2.52]	1.00 [0.43–2.35]
**BMI category**, n (%)		
Underweight	Reference	Reference
Normal	1.06 [0.42–2.64]	0.85 [0.31–2.32]
Overweight/obese	2.20 [0.50–9.61]	1.43 [0.29–6.94]
**IRP4s < 5 mm Hg**, n (%)	**2.59 [1.34–4.97]**	**2.21 [1.12–4.37]**
**Esophagogastric junction type**, n (%)		
Type I	Reference	Reference
Type II	2.12 [0.75–5.96]	2.31 [0.79–6.80]
Type III	0.91 [0.39–2.10]	0.89 [0.36–2.18]

BMI = body mass index, CI = confidence interval, IRP4s = 4-second integrated relaxation pressure, OR = odds ratio.

## 4. Discussion

The most important finding in this study is the presence of absent contractility in patients without rheumatologic diseases. Most patients had reflux or dysmotility symptoms, which are also the manifestations of GERD. One-half of the patients studied had erosive esophagitis, mainly of a mild grade. Low IRP4s, suggesting the weak capacity of LES relaxation, was associated with erosive esophagitis on endoscopy as was male sex.

### 4.1. Prevalence of absent contractility

In this study, only 2.7% of the patients who underwent HRM were diagnosed with absent contractility. This finding confirms the results of previous studies that this condition is an uncommon esophageal motility disorder (4% in patients with all indications for HRM, 3% in patients with GERD, and 5%–7% in patients with dysphagia).^[13–16]^

While absent contractility is often studied in the scope of rheumatologic diseases, especially systemic sclerosis, no patients in our study had a history of these disorders or suspected symptoms.^[2–4,17]^ In a study of 207 patients with absent contractility, nearly two-thirds had systemic sclerosis, 20% had another systemic condition, and only 16% had non-rheumatologic disease.^[2]^ One explanation is that our center did not receive patients with systemic disease from rheumatologists or dermatologists, but primarily patients who presented with dysphagia, extraesophageal reflux symptoms, or refractory GERD. Since the prevalence of systemic autoimmune diseases is very low,^[18,19]^ and these patients often visit a specialized center rather than a general medical clinic, it is uncommon to observe patients with rheumatologic diseases in our clinic without a referral from other specialists. Our findings suggest that absent contractility may not be limited to individuals with rheumatologic disease and may be more common in the general population than has been previously thought. Furthermore, so far, it has been unclear which one between GERD and hypomotility disorders is the cause and which one is the effect.^[20,21]^ In the Chicago Classification version 4.0, the diagnosis of absent contractility may not be clinically relevant if patients do not have significant dysphagia, weight loss or reflux symptoms or HRM is done prior to antireflux surgery.^[22]^ Additionally, absent contractility could be the “early phase” of achalasia type I, especially when IRP is around the normal cutoff. In these cases, other tests such as rapid drink challenge or solid meal challenge during HRM, barium swallow or FLIP should be performed to find or exclude obstructive findings.

On the other hand, it is necessary to evaluate the motility function of patients with systemic disease, especially systemic sclerosis, because these patients might have esophageal dysmotility, which may be associated with interstitial lung disease.^[3,23,24]^ In a 2019 study at the Vietnam National Hospital of Dermatology and Venereology, among 57 patients diagnosed with systemic sclerosis, 86.0% and 42.1% reported reflux symptoms and dysphagia, respectively.^[25]^ In a systematic review to evaluate the role of HRM to detect therapeutic effects in patients with systemic sclerosis, the result showed HRM is a highly bothersome technique, but has uncertain impact on significant improvement in patient care; and not superior than validated scores in assessing clinical burden in the follow-up.^[23]^ However, HRM has an important role among patients with the indication of lung transplantation. Lung transplantation is a therapeutic option for patients with end-stage lung disease due to systemic sclerosis and needs a carefully selection.^[26]^ A few studies addressed absent contractility is a relative contraindication for this intervention.^[27]^ More research is needed to identify the utility of HRM in the management of patients with systemic diseases, especially in the cases with severe symptoms of reflux or difficult swallowing.

### 4.2. Clinical manifestations

Our study showed a high prevalence of GERD-like symptoms (heartburn, regurgitation, and high GERD scores) among patients with absent contractility. The high prevalence of heartburn and regurgitation was similar to a study of 66 patients who had absent contractility at a tertiary referal center in the United States.^[28]^ Sixty-two percent of patients in this study had pathological (acid exposure time > 6%). This study also reported a high number of patients who experienced dysphagia (47.1%) as ours (32.4%).^[28]^ Dysphagia is a common symptom of functional or mechanical esophageal obstruction and dysmotility.^[29]^ Thus, modern diagnostic techniques such as HRM could help explore underlying conditions and determine the management strategy for each individual.

### 4.3. Prevalence of erosive esophagitis

Few publications on absent contractility have determined the prevalence of erosive esophagitis. In our study, 50% of patients with absent contractility had erosive esophagitis on endoscopy, similar to a study in 66 American patients with absent contractility (57.7%).^[28]^ Although absent contractility is an independent factor of erosive esophagitis,^[30,31]^ the prevalence of esophagitis in our study was similar to previous studies observed in GERD populations (37%–65%).^[32,33]^ The absence of esophageal body contraction could predispose and worsen GERD complications.^[20,21]^ PPIs and H2-receptor antagonists are two effective medication therapies in healing erosive esophagitis.^[34]^ Prokinetic agents are expected to improve esophageal muscle contractility and motor function, however the effects of theses medicines on absent contractility are still limited. Prucalopride and mosapride are considered as first-line prokinetic agents in the treatment of absent contractility due to their impact on peristaltic amplitude. In our study, more than one-third of patients used reflux symptoms medications (PPIs, H_2_-receptor antagonists, antacids) and 9 patients were prescribed with domperidone and itopride within the last 3 months before diagnosing with absent contractility. However, no study has yet explored the changes in esophageal motility before and after treating esophagitis and factors associated with the improvement of esophageal motility over time.

### 4.4. Factors associated with erosive esophagitis

In our study, male sex and low IRP4s (<5 mm Hg) were associated with erosive esophagitis. These factors are also considered risk factors among patients with GERD.^[21,35–38]^ Studies have highlighted the predominance of males in pathological changes related to reflux (including erosive esophagitis, Barrett’s esophagus, and esophageal adenocarcinoma). In one experimental study, testosterone was found to correlate with the number and function of parietal cells which enhanced the gastric secretion while female sex hormones had inhibitory effect.^[39]^ It was also confirmed in the later clinical study with the 24-hour pH monitoring reporting significantly longer acid exposure time in males comparing to females. The more vulnerable mucosal integrity in males were explained by the protective mechanism of estrogen in females.^[40]^ Furthermore, the social habits such as smoking, alcohol consumption with predominant prevalence in males were classified as risk factors of more severe esophagitis and occurrence of Barrett’s esophagus and adenocarcinoma.^[40–42]^

Esophagogastric junction tone is an important gastroesophageal barrier to prevent reflux. Therefore, the failed peristalsis in the setting of absent contractility, when combined with esophagogastric junction hypotension, could increase esophageal acid burden,^[43]^ thus increasing the risk of esophagitis. However, it remains unknown whether absent contractility worsens GERD or the inflamed esophageal mucosa in reflux esophagitis results in failed peristalsis.^[13,20,31]^

### 4.5. Study strengths and limitations

Our findings should be interpreted with appropriate caution. First, this is a single-center study done with convenience sampling, so the prevalence of absent contractility observed in this study might not be generalizable to the Vietnamese general population. Second, due to limited resources, we only used collected self-reported data on the past history and clinical examination to exclude systemic diseases. This might have resulted in missing cases with atypical or early phase presentations. However, to our knowledge, this study included one of the largest populations of patients with absent contractility, especially in Asian population. The finding that absent contractility was also present outside the domain of rheumatologic diseases is new.

## 5. Conclusions

Absent contractility was present in many patients without known systemic diseases. Erosive esophagitis was common and associated with male sex and low IRP4s.

## Author contributions

**Conceptualization:** Long Van Dao.

**Data curation:** Long Bao Hoang.

**Formal analysis:** Long Bao Hoang.

**Funding acquisition:** Hang Viet Dao.

**Investigation:** Hang Viet Dao.

**Methodology:** Hang Viet Dao, Long Bao Hoang.

**Supervision:** Hue Thi Minh Luu, Hoa Lan Nguyen, Robert Joel Goldberg, Jeroan Allison, Long Van Dao.

**Validation:** Hang Viet Dao, An Thi Minh Dao, Hong Thi Van Nguyen, Tomoaki Matsumura, Long Van Dao.

**Writing – original draft:** Hang Viet Dao.

**Writing – review & editing:** Hang Viet Dao, Long Bao Hoang, Hue Thi Minh Luu, Hoa Lan Nguyen, Robert Joel Goldberg, Jeroan Allison, An Thi Minh Dao, Hong Thi Van Nguyen, Tomoaki Matsumura, Long Van Dao.
